# Faint of Heart: A Case of Atrial Myxoma Presenting With Atypical Syncope

**DOI:** 10.7759/cureus.39935

**Published:** 2023-06-04

**Authors:** Manisha Koneru, Kyle Gleaves, Satyajeet Roy

**Affiliations:** 1 Internal Medicine, Cooper Medical School of Rowan University, Camden, USA; 2 Internal Medicine, Cooper University Health Care, Camden, USA

**Keywords:** cardiac mass tumor, atrial myxoma, cardiogenic syncope, benign cardiac mass, unexplained syncope, transthoracic echocardiogram

## Abstract

Syncope is a frequent reason for elderly patients to present to an outpatient office. The underlying cause of syncope can range from benign to serious. Although serious cases of syncope are rare, appropriate workup can help detect and address potentially fatal pathologies. We present a case of a 74-year-old woman who presented with an episode of syncope with associated epigastric cramping. Sudden syncope without significant comorbidities prompted further diagnostic workup, which revealed a rare cardiac myxoma. This case underscores the importance of ruling out potentially fatal causes prior to favoring more conservative diagnoses when investigating syncope in the elderly population.

## Introduction

Syncope in the elderly population can have multiple etiologies, ranging from relatively benign to lethal [1]. Common causes of syncope include vasovagal and orthostatic changes. Other cardiovascular, neurologic (i.e., transient ischemic attack), endocrinologic, and psychiatric etiologies may also present as syncopal episodes [1,2]. Overlap in presentation characteristics among a wide variety of potential syncope causes poses a diagnostic challenge to clinicians in an outpatient setting.

Cardiogenic syncope is caused by heart abnormalities that prevent sufficient cerebral circulation and thus deprive the brain of the appropriate oxygenation required for consciousness [3]. The underlying etiology may be structural or electrical, facilitating aberrant electrical activity [1-3]. Epidemiologically, cardiogenic syncope is most prevalent in patients over the age of 60 years; 30% of syncopal episodes are of cardiac etiology in this subpopulation [3]. Its prevalence is also higher in patients with comorbid diabetes mellitus, hypertension, or hyperlipidemia [2]. Cardiogenic syncope is typically indicative of potentially serious underlying pathology, with a one-year mortality rate of 30% [3]. Thus, it is imperative to appropriately investigate potential cardiac pathology in the diagnostic evaluation for syncope, particularly when co-presenting with atypical symptoms.

This case was previously presented as a meeting abstract and poster at the 10th Annual Camden Scholars Forum on May 3, 2023.

## Case presentation

A 74-year-old woman with a relevant history of hypertension, hyperlipidemia, and asthma well-managed with lifestyle interventions presented to our outpatient office following a fainting episode. She was singing in a choir when she developed a cramp-like discomfort in the epigastric region followed by loss of consciousness. Witnesses reported that her eyes had rolled back into her head, but denied any tongue-biting, urinary incontinence, or convulsions. She regained consciousness within a few seconds, but she was noted to be confused and diaphoretic afterward. She returned to her baseline within a few minutes.

The patient endorsed some residual lightheadedness but denied fever, chest pain, palpitations, headache, nausea, or shortness of breath. One week prior to the presentation, she had experienced an episode of vertigo. Her physical exam was notable for hypertension and a unilateral erythematous tympanic membrane. She had a negative Dix-Hallpike maneuver. The orthostatic supine-to-standing test resulted in only a 10 mmHg drop in systolic blood pressure. The rest of her vital signs and physical examination findings were within normal limits.

Her recent episode of vertigo was attributed to an acute otitis media, for which she was treated with a 10-day course of twice daily oral amoxicillin-clavulanate antibiotic. Given her co-presenting epigastric pain with syncope, the differential diagnosis for the syncopal episode was expanded to include vasovagal syncope, cardiogenic syncope, sick sinus syndrome, seizure, or myocardial infarction.

Her in-office electrocardiogram showed normal sinus rhythm with no other abnormalities, which indicated that sick sinus syndrome and recent myocardial infarction were less likely. No tongue-biting, urinary incontinence, myoclonic movements, post-ictal state, and prior history indicated seizures were not a likely cause of the episode. Additional diagnostic tests, such as a complete metabolic panel, complete blood count, and chest radiograph were within normal limits.

**Figure 1:**
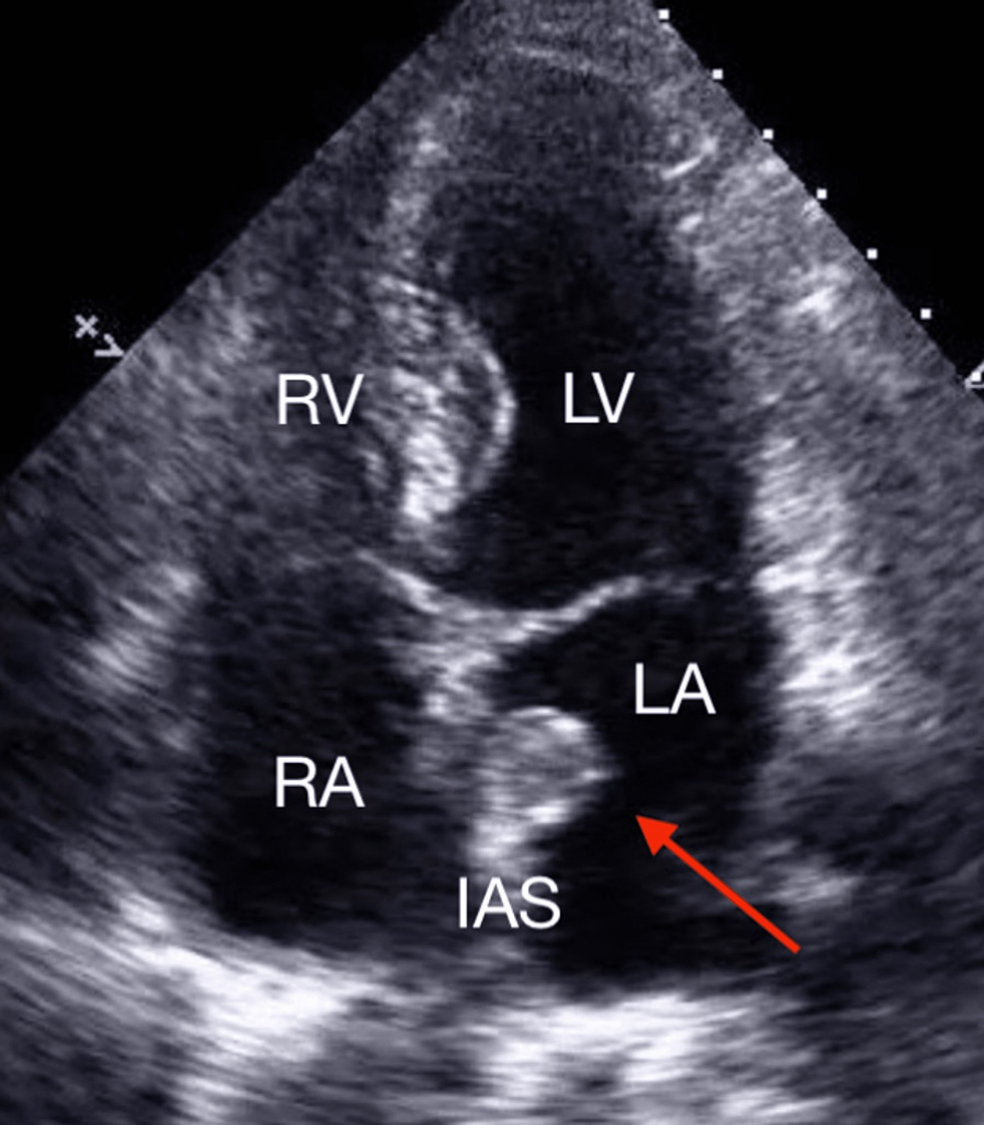
Apical Four-Chamber View of Atrial Myxoma Transthoracic echocardiogram showing a 2x2.3 cm heterogenous mass (arrow) protruding from a thickened interatrial septum into the left atrium RA = right atrium; LA = left atrium; RV = right ventricle; LV = left ventricle; IAS = interatrial septum

**Figure 2:**
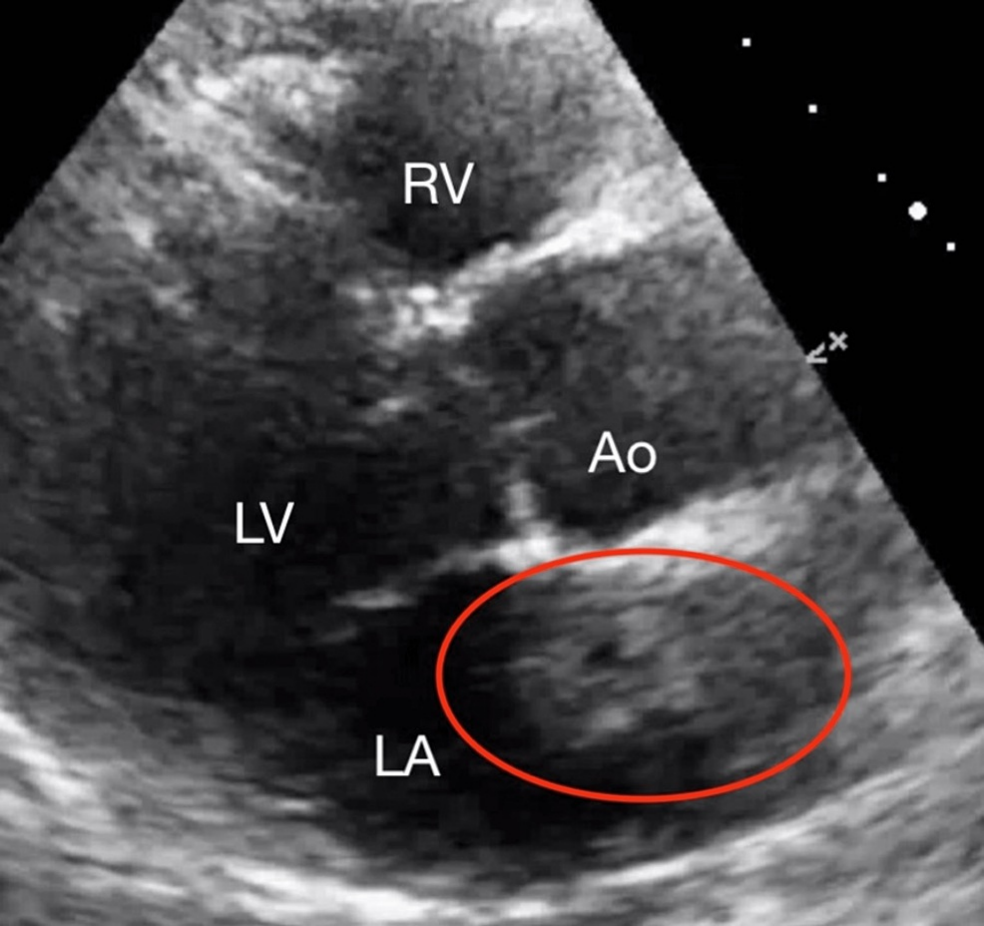
Parasternal Long-Axis View of Atrial Myxoma Transthoracic echocardiogram demonstrating a partially obstructive mass (circled) tethered to the interatrial septum LA = left atrium; LV = left ventricle; RV = right ventricle; Ao = aorta

**Figure 3:**
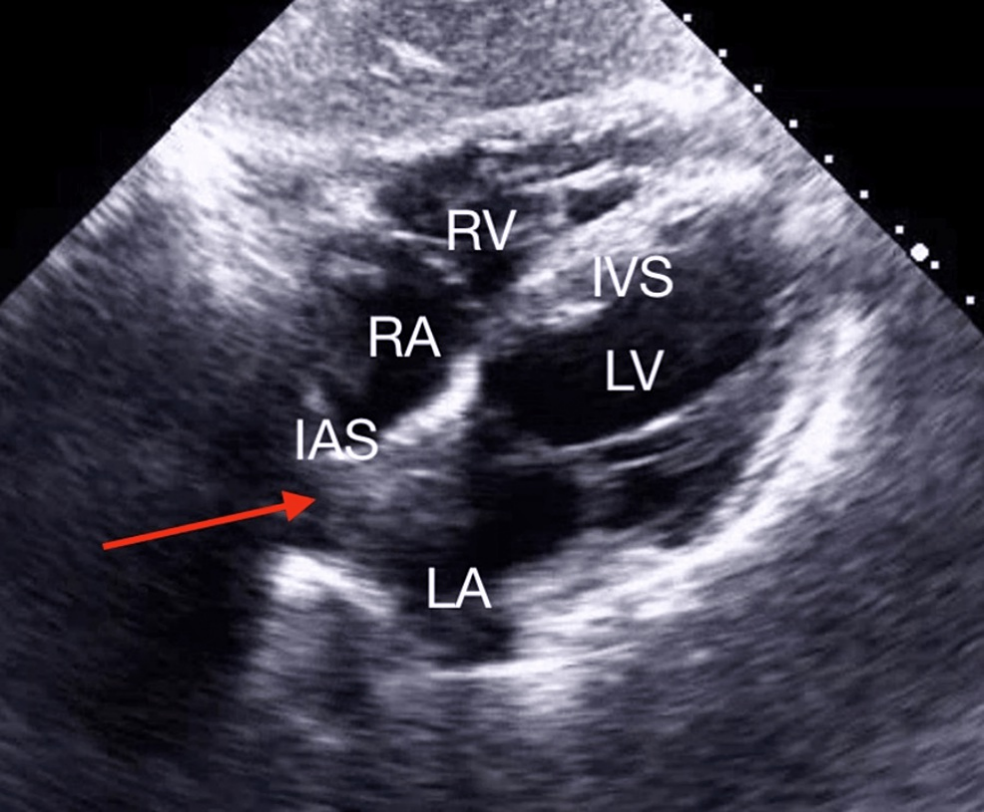
Subcostal Chamber View of the Atrial Myxoma Transthoracic echocardiogram showing mass (arrow) protruding into left atrium causing partial obstruction during systole. RA = right atrium; LA = left atrium; RV = right ventricle; LV = left ventricle; IAS = interatrial septum; IVS = interventricular septum

A transthoracic echocardiogram (TTE) was performed to examine structural cardiac pathology before favoring a diagnosis of vasovagal syncope. The TTE demonstrated a 2x2.3 cm, non-mobile, heterogenous mass in the left atrium tethered to a thickened interatrial septum (Figures 1-3). This mass was partially obstructive, as it would slow systolic blood flow without evidence of full occlusion on Doppler echocardiography. This mass was suspected to be an atrial myxoma (AM).

Although the mass is relatively small, the obstruction of forward blood flow likely was exacerbated to a symptomatic degree due to hypovolemia. At the time of the syncopal episode, she was engaging in physically demanding activity (i.e., singing) in a warm room, which contributed to a dehydrated state. Consequently, the patient's volume status was likely key to why an otherwise silent cardiac mass was able to induce a transient flow obstruction.

She underwent an excision of the atrial mass. The pathology findings confirmed the diagnosis of AM. Grossly, the mass had evidence of hemorrhagic components and calcifications. Microscopically, there were polygonal cells forming cords scattered within a myxoid stroma. Cells were positive for cytoplasmic calretinin and S100 but were negative for SMA, desmin, CD68, and cytokeratins.

Her postoperative course was complicated by the development of tachycardia-bradycardia syndrome, which required a permanent pacemaker placement. Subsequently, she remained asymptomatic and was medically managed long-term with metoprolol succinate, rosuvastatin, and rivaroxaban.

## Discussion

AM is a benign cardiac mass; approximately 50% of primary cardiac tumors are AM [4]. AM is estimated to occur in less than 1% of the total population [4]. A definitive diagnosis occurs primarily through gross and microscopic pathology [4,5]. Thus, most confirmed diagnoses occur with pathology following surgical intervention or during autopsy [5]. Curative treatment is with surgical excision of the mass [4]. Recurrence in nonhereditary atrial myxoma is seen in less than 3% of cases [6]. The clinical manifestation of AM is dependent on tumor size [5]. The presentation can range from being completely asymptomatic to having moderate, non-specific obstructive cardiopulmonary symptoms, to sudden death depending on the degree of obstruction and impact on forward flow dynamics [5].

In our patient, syncope was the chief presenting symptom. This case demonstrates the diagnostic challenge while identifying AM, given its nonspecific stigmata within the larger possibilities for syncope and other mimics, including vasovagal, cardiogenic, orthostatic, neurologic, endocrinologic, pharmacological, hematological, and psychiatric causes [1-3,5]. However, the potential to precipitate fatality underscores the importance of pursuing appropriate diagnostic testing when accompanying atypical symptoms, such as epigastric pain, do not fit into the conventional presentations for more common and conservative diagnoses of syncope such as vasovagal and orthostatic syncope.

## Conclusions

In conclusion, syncope is a common chief complaint for elderly patients presenting to an outpatient office. Syncope, particularly in patients co-presenting with atypical symptoms, such as epigastric cramping, should prompt further diagnostic testing to evaluate for potentially life-threatening cardiac etiology before favoring more conservative diagnoses such as vasovagal syncope. This case of syncope in an otherwise healthy elderly patient ultimately diagnosed as atrial myxoma illustrates the significance of maintaining a broad differential diagnosis when characterizing a common, non-specific symptomatic presentation to ensure potentially fatal etiologies have been adequately evaluated for and ruled out.
